# From quantum communication fundamentals to decoherence mitigation strategies: Addressing global quantum network challenges and projected applications

**DOI:** 10.1016/j.heliyon.2024.e34331

**Published:** 2024-07-11

**Authors:** Muhammad Annas Khan, Salman Ghafoor, Syed Mohammad Hassan Zaidi, Haibat Khan, Arsalan Ahmad

**Affiliations:** a SEECS, National University of Sciences and Technology (NUST), H-12, Islamabad, Pakistan; b Ghulam Ishaq Khan Institute of Engineering Sciences and Technology, Pakistan; c College of Aeronautical Engineering, National University of Sciences and Technology (NUST), H-12, Islamabad, Pakistan

**Keywords:** Quantum network, Quantum communication, Quantum teleportation, Quantum error correction codes

## Abstract

In the aftermath of unparalleled disruptive technologies, the quantum realm has become a fundamental field of research due to unrivaled computational power and super-secure communication. In addition to conventional networks, a new word in the quantum domain is quantum network. The quantum network uses quantum communication (QC) to send quantum information bits known as qubits, to predetermined destination nodes. It governs the new quantum mechanics notions like superposition, quantum entanglement, the no-cloning theorem, and quantum teleportation. Quantum communication, like classical communication, is prone to noise, which is known as quantum decoherence. Quantum decoherence is a significant barrier to the implementation of a global quantum network. It deteriorates the quantum information, causing it to lie in an undetermined state. Environmental factors that cause quantum entanglement loss are the key factors that cause qubits to lose their states. To mitigate the impact of quantum decoherence, quantum error correction codes (QECC) and entanglement distillation have proved their potential. They add extra qubits or maintain entanglements among quantum networks. This survey presents quantum mechanics principles, quantum decoherence, and techniques to mitigate the effect of quantum decoherence. At the end, we highlighted some challenges in the realization of the quantum network, along with some projected applications.

## 1 Introduction

Since the start of the 21st century, significant efforts have been made to advance reliable quantum applications, particularly in Quantum Computing, Quantum Communication (QC) [1], Quantum Cryptography, and Quantum Internet (QI) [2]. Quantum computing, in particular, has gained widespread attention in recent years due to its exceptional computational power, with major tech companies such as IBM, Google, Microsoft, and Intel actively competing to lead in this field [3]. Their primary goal is to enhance computing power by increasing the number of qubits, as a higher qubit count leads to immense computational capabilities in quantum processors. For instance, in 2017, Google unveiled “Sycamore,” a cutting-edge 54-qubit quantum processor [4]. Subsequently, in 2021, IBM achieved a significant milestone by surpassing its predecessors with a 100-qubit processor [5]. This increasing potential in quantum computing has attracted considerable investments, with venture funding in the quantum computing sector reaching approximately 25 billion in 2021 [6]. Notably, both the USA, EU, and various companies have initiated flagship projects to excel in this promising domain. Table 1 provides an overview of various global strategies and plans for quantum networks (QNs), showcasing the substantial amount of work conducted over the past two decades to establish fully functional quantum networks. Unfortunately, Quantum networks face a major hindrance in the form of quantum channel noise known as quantum decoherence which disrupts the fragile quantum states during transmission, hindering accurate quantum information exchange and compromising the integrity of quantum communication. This susceptibility to decoherence complicates the establishment of reliable and long-distance quantum communication links, underscoring the necessity to address this fundamental issue for the successful deployment of quantum networks.

**Table 1:** List of some national plan/strategy/networks.

Year	Nation	Plans/Strategies/Networks
2022	Singapore	National Quantum-Safe Network in the Quantum Engineering Program
2022	USA	The Quantum Cybersecurity Preparedness Act
2021	UK	The European Quantum Communication Infrastructure (EuroQCI) Initiative
2019	UK	Cambridge Quantum Network
2010	Japan	Tokyo QKD network
2009	China	Chinese Hierarchical Network, Wuhu
2003	Austria	SECOCQ QKD Network

**Figure 1:**
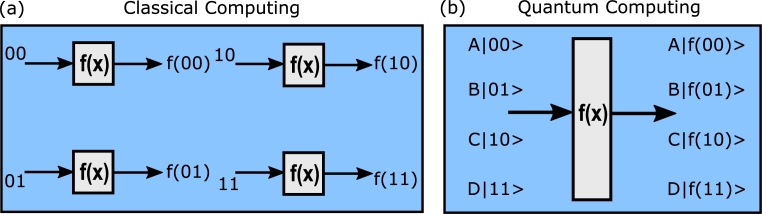
Computational power illustration between (a) classical and (b) quantum computing [21].

In contrast to the Classical network, Quantum Network (QN) allows for the secure and dependable transfer of quantum information (Qubits) among quantum devices [7], [8]. Qubits can exist in a superposition of both 0 and 1 states simultaneously, depending on probabilities. This unique property enables the attainment of significant computational power. As depicted in Fig. 1, while two classical bits can produce four possible outcomes, a single qubit achieves the same. Essentially, a single qubit can encode the same amount of data as two classical bits. This relationship follows a pattern of $2^{n}$ power for qubits. Consequently, an increased number of qubits results in an exponential rise in the encoded data capacity.

In the early days of quantum mechanics, pioneers like Niels Bohr, Werner Heisenberg, and Erwin Schrödinger laid the foundation for quantum theory. They introduced the concepts of superposition and wave-particle duality and the measurement problem, which led to questions about quantum reality and observers' roles. John von Neumann introduced the density matrix formalism, which played a crucial role in the development of quantum decoherence theory. During the 1970s, researchers explored “many-worlds” interpretations of quantum mechanics, with decoherence playing a crucial role in explaining wave function collapse. During the 1990s, experiments showed the effects of decoherence on quantum systems, with researchers studying superconducting circuits, trapped ions, and quantum dots. The development of quantum information theory and quantum computing has highlighted the impact of decoherence on quantum systems, with researchers working on error correction and fault-tolerant computation to mitigate its effects. Understanding decoherence has practical implications for quantum technologies such as quantum computing, communication, and sensing, which rely on controlling and minimizing its effects for reliable and scalable quantum operations [9] [10] [11] [12] [13] [14].

**Figure 2:**
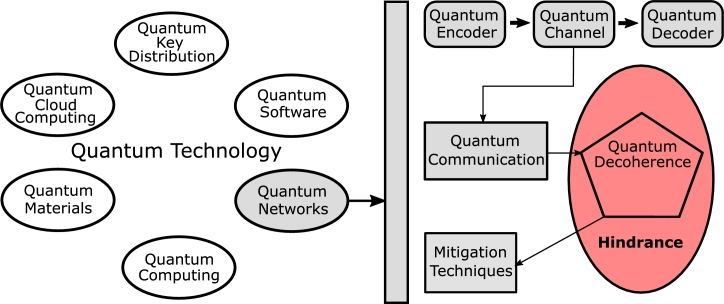
Quantum technology overview.

The world of quantum mechanics offers the promise of secure and high-speed communication. However, it is also susceptible to environmental noise, which can introduce imperfections in quantum hardware and cause qubits to lose their state. This phenomenon is known as quantum decoherence, a form of quantum noise that lacks a classical counterpart. Overcoming these imperfections and realizing global quantum communication requires extensive research and investigation [15]. Various methods are being explored to mitigate the effects of quantum decoherence. Some of these include Quantum Error Correction Codes (QECC) [16], which aim to detect and correct errors that occur during quantum computation or communication. Entanglement distillation or purification techniques [17] are utilized to enhance the quality of entangled qubits, thereby improving their resilience to decoherence. Additionally, fault-tolerant techniques [18] are developed to ensure reliable quantum computations even in the presence of errors. To comprehensively understand and implement these mitigation techniques, it is essential to have a complete grasp of basic quantum mechanics principles and their mathematical descriptions. This foundational knowledge provides an overview of the strategies to counter the challenges posed by quantum decoherence and facilitates advancements in quantum communication. The fast-developing discipline of quantum technology uses the ideas of quantum physics to create new systems that are more capable than traditional ones. Technologies include quantum software, quantum cryptography, quantum computing, and quantum networks, as depicted in Fig. 2.

This paper aims to shed light on the crucial concepts of quantum mechanics along with the following objectives:

• To overview the quantum mechanics preliminaries along with Quantum communication, whose major hindrance is quantum decoherence.
• Study the concept of Quantum Decoherence and compare its mitigation techniques. Based upon these techniques, we propose a future direction feasible for the practical realization of QN.

**Figure 3:**
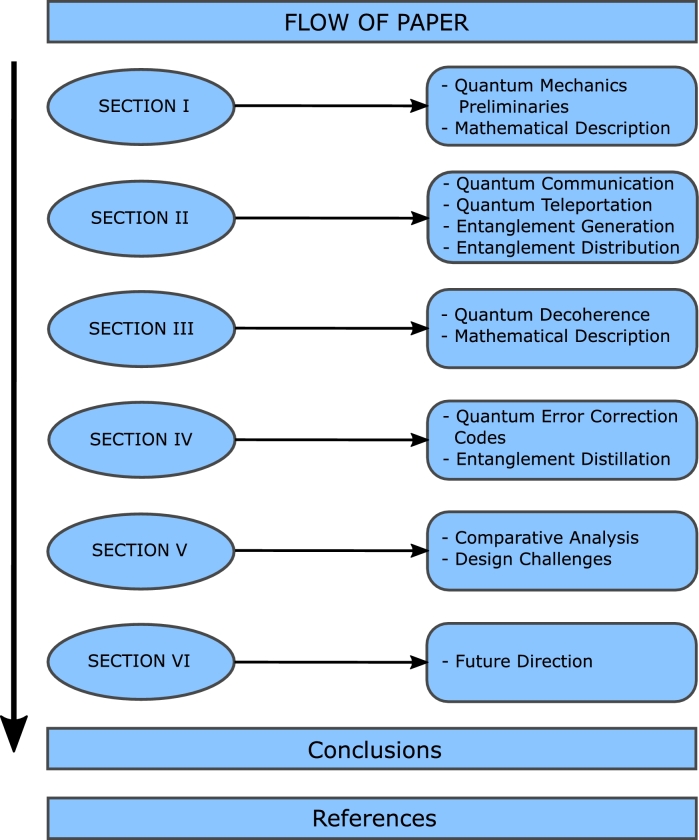
Flow of the paper.

The paper's flow is outlined in Fig. 3. It consists of several sections, each covering distinct aspects related to quantum mechanics, quantum communication, quantum decoherence, mitigation techniques, and challenges in achieving a Quantum Network (QN). Section 1 will delve into the key concepts of quantum mechanics to establish the groundwork for the rest of the paper. Section 2 presents an overview of quantum communication. Section 3 discusses the theoretical and mathematical background regarding quantum decoherence. This section will shed light on the effects of environmental noise and imperfections on qubits. In Section 4, the techniques proposed by the research community on Quantum Error Correction Codes (QECC) and entanglement distillation will be reviewed. These approaches aim to mitigate the impact of quantum decoherence. In Section 5, a comparative analysis of the different mitigation techniques will be conducted, along with an examination of the challenges faced in achieving QI. This includes exploring flaws in the quantum teleportation process, quantum-classical synergy, hardware limitations, and joint modeling of quantum imperfections. Section 6 will conclude the paper, providing a discussion of future perspectives and potential directions for further studies in pursuit of a global Quantum Network (QN).

## 2 Quantum mechanics: preliminaries and mathematical description

Quantum applications such as QI works on the principles of quantum mechanics. Quantum mechanics principles that mainly govern the in-depth technology of QI are superposition, Entanglement, and No-cloning theorem. This section will explain the qubit and its generation methods along with the main quantum principles.

### 2.1 Qubits

Quantum bits or qubits are the quantum information having two measurable states mainly called as 0's or 1's [19]. With two orthogonal basis states, denoted by |0〉 and |1〉, respectively, it has two discrete quantum levels. |0〉 is called as ground state while |1〉 is called as the excited state. Unlike classical bits whose values at a time completely lie on either 0 or 1, qubits have a value between 0 and 1. It is important to note that once it is measured, it collapses to one of its orthogonal bases. Qubit is described by state vector as:

(1) |ψ〉=α|0〉+β|1〉

**Figure 4:**
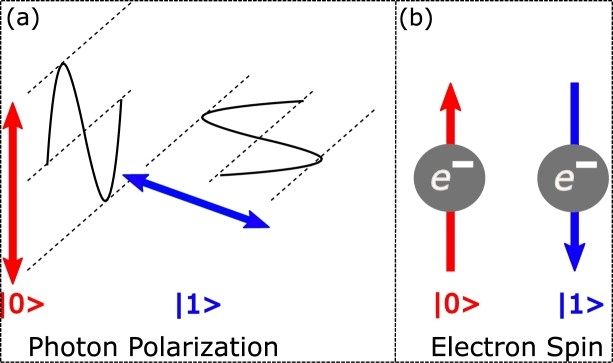
Physical implementation of Qubits. Representation of state 0 and 1 through (a) Photon polarization or through (b) Electron spin.

Where |〉 is known as Ket or Dirac notation. Qubits are generated physically using various methods. Different applications such as quantum communication, quantum sensing, and quantum computing use different implementations of qubits. As the photonic implementation of qubits using photonic devices is getting mature, this area has advanced at a rapid rate [20]. Illustration of qubit via photon is performed through the polarization of the photon. Vertical polarization can be represented as |1〉 and horizontal polarization as |0〉. A qubit can also be represented by an electron, whose states are represented as electron spin. When light shines on an atom for a certain period of time, it is possible to move the electron from the ground to an excited state. |0〉 represents “spin up” while |1〉 represents “spin down”, as depicted in Fig. 4. Each implementation is accomplished through various techniques.

### 2.2 Superposition

**Figure 5:**
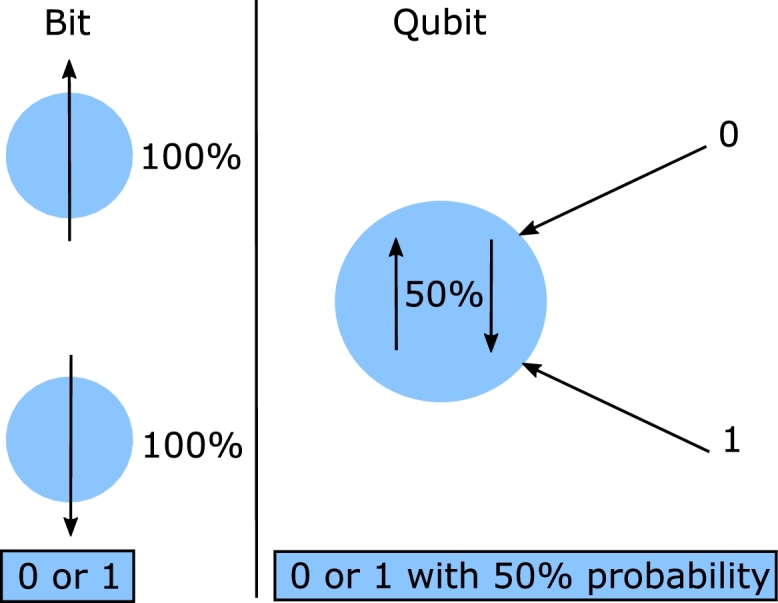
Superposition principle.

Classical mechanics provides a comprehensive understanding of position and momentum. However, quantum mechanics introduces the principle of superposition, which explains how quantum entities like qubits can exist in a combination of two states. A qubit can be in a state of both 0 and 1 simultaneously [21]. It maintains this superposition until it is measured. Once measured, the qubit collapses into either the state 0 or 1. This measurement outcome is classical information, causing the quantum system to transition into the classical domain. A famous thought experiment, Schrödinger's cat, exemplifies the superposition principle. In this experiment, the cat is considered to be simultaneously both dead and alive, based on a random event that may or may not occur [22]. Fig. 5 illustrates the concept of superposition.

### 2.3 Entanglement

**Figure 6:**
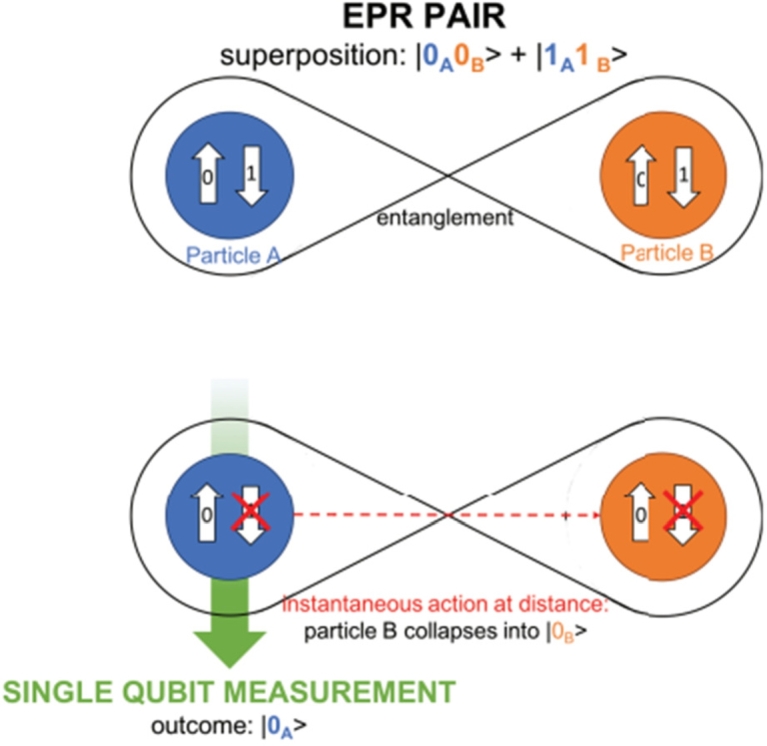
Entanglement principle [24].

Entanglement is a mysterious property that connects two particles even when they are physically separated by a certain distance. The wave function describing the entangled particles cannot be independently defined for each particle, meaning that the measurement of one particle will instantaneously affect the other, regardless of the distance between them [23]. In other words, the quantum state cannot be factored into individual states for each particle, making them inseparable. The information of one particle is not fully determined until information about the other particle becomes available. Fig. 6 illustrates this instantaneous correlation between entangled particles [24]. This unique phenomenon of entanglement has no classical counterpart and plays a fundamental role in many quantum applications. When a group of N qubits is superposed and entangled together, they can collectively encode information in a 2N-dimensional Hilbert space. This property leads to exceptional computational power in quantum applications.

### 2.4 No-cloning theorem

Classical communication possesses the ability to copy, retransmit, and measure classical bits at any point in time. It employs various routing protocols to perform different network functionalities effectively. In contrast, quantum communication lacks these abilities due to the quantum nature of qubits. According to the no-cloning theorem, it is impossible to accurately measure or duplicate a quantum state [25]. Consequently, during the transmission period, a qubit cannot be measured, as doing so would cause it to collapse into a classical bit. This restriction prevents the use of classical error correction schemes in quantum communication. To securely transmit qubits from one end node to another without compromising their quantum properties, Quantum Error Correction Codes (QECC) are applied. These codes enable the detection and correction of errors that may occur during quantum communication, ensuring the reliable and secure transfer of quantum information. By utilizing QECC, quantum communication can maintain the delicate quantum states of qubits while safeguarding against noise and decoherence in the transmission process.

## 3 Quantum communication

Within the paradigm of quantum mechanics, quantum communication and quantum decoherence are closely related. To communicate information securely and effectively, quantum communication makes use of the concepts of entanglement and superposition as discussed in section 1. Quantum decoherence, which occurs when quantum states are particularly sensitive to external interactions, is caused by these precise principles. A fundamental conflict arises from the delicate balancing act between using quantum mechanics to communicate and reducing the disruptive consequences of decoherence. To preserve the dependability and integrity of transmitted quantum information, successful quantum communication systems must carefully manage this dynamic, leveraging quantum processes while regulating and minimizing the influence of decoherence.

**Figure 7:**
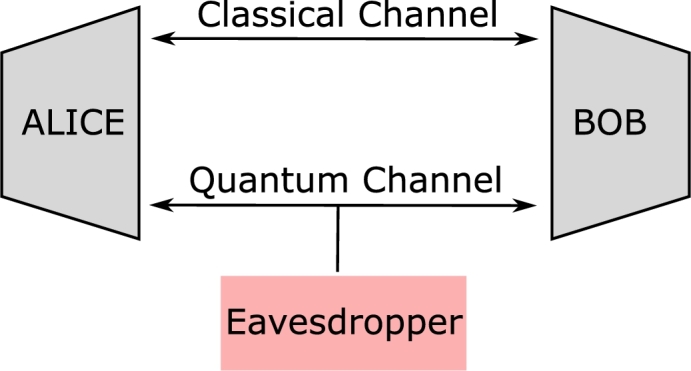
Quantum Communication Model.

Communication through a quantum channel faces limitations imposed by classical information theory. Therefore, there arises a necessity to interpret classical information theory within the domain of quantum perception [26]. Quantum communication (QC) serves as the means to transfer quantum information, represented by qubits, in an encoded manner from one node to another, exploiting the peculiar laws of quantum mechanics [27]. Its fundamental purpose is to enable qubits to teleport their states via a quantum channel. A remarkable feature of quantum communication is its ability to provide enhanced security against eavesdroppers. Just as classical communication is affected by noise, quantum communication is also susceptible to a phenomenon known as quantum decoherence. It is important to note that classical communication and quantum communication work in parallel to achieve the complete realization of Quantum Information (QI). The core strategy behind quantum communication lies in quantum teleportation, which bestows it with high security and rapid communication capabilities. A basic quantum communication model involves the utilization of both classical and quantum computers at the transmission ends, labeled as ALICE and BOB, respectively, along with an eavesdropper positioned at the quantum channel, as illustrated in Fig. 7.

### 3.1 Quantum teleportation

Quantum teleportation and quantum decoherence are interlinked through their fundamental impact on the reliability of transmitting quantum information [28]. Quantum teleportation enables the transfer of quantum states between distant particles, a feat achieved by entangling the sender and receiver particles and utilizing classical communication to recreate the state [29]. However, quantum decoherence, arising from interactions with the environment, poses a significant challenge to maintaining the integrity of the transmitted quantum state. As the fidelity of the teleported state is compromised by decoherence effects, strategies like entanglement distillation and error correction become essential to counteract these losses. Therefore, the success of quantum teleportation hinges on mitigating quantum decoherence and emphasizing their intimate interconnection in the quest for robust quantum communication and computation [30].

**Figure 8:**
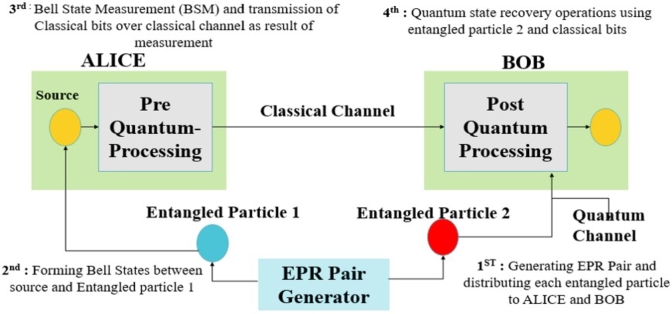
Quantum Teleportation.

The fundamental principle of Quantum Information (QI) revolves around transmitting quantum information between quantum end nodes. To achieve this objective, Quantum Teleportation (QT) was introduced in [31]. QT facilitates the transmission of quantum information without directly sending the actual quantum state; instead, it transmits the state of qubits. This process relies on two interconnected channels: a quantum channel and a classical channel. The classical channel transmits the two classical bits resulting from the collapse of qubits after measurement. Concurrently, the quantum channel transmits the entangled qubits known as EPR pairs (Einstein-Podolsky-Rosen pairs). These EPR pairs derive their name from a famous paper by Einstein [32]. Ensuring a strong integration between the quantum and classical channels is crucial. This is because, upon measurement, entanglement is lost between the EPR pairs, necessitating the use of new EPR pairs to teleport the quantum information. These EPR pairs are generated using various techniques, commonly referred to as EPR pair generators. Fig. 8 illustrates the setup of Quantum Teleportation (QT) along with its corresponding block diagram, showcasing the interplay between the quantum and classical channels.

**Figure 9:**
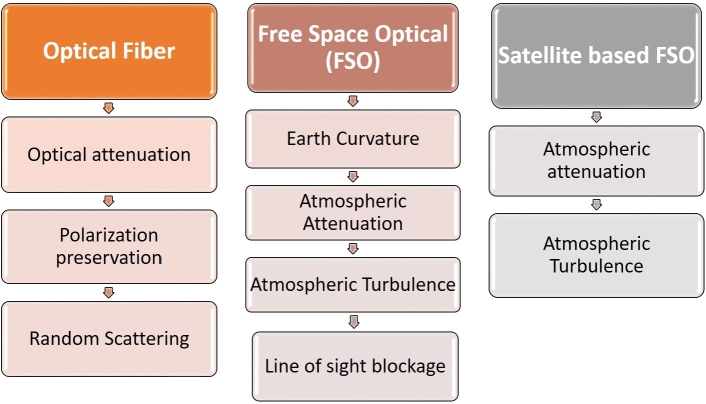
Quantum channel comparison.

Quantum teleportation mainly consists of two steps mainly known as Entanglement generation and Entanglement distribution [33]. As Quantum decoherence undermines the fidelity of quantum information transmission through quantum channels, this is the main area of interest to discuss. Three scenarios are employed for EPR pair distribution: optical fiber, free space optics (FSO), and satellite-based FSO [34]. Quantum decoherence reduces the distance of quantum teleportation. Satellite-based FSOs are less prone to decoherence and possess more adaptable infrastructure, resulting in greater communication range. Optical fiber, on the other hand, has higher losses and is less adaptable to infrastructure, resulting in shorter communication distances as compared to satellite-based FSO. Fig. 9 shows the comparison between these three scenarios in terms of losses.

## 4 Quantum decoherence

This section discusses the most common challenge for qubits which is quantum decoherence. Indeed, one of the crucial challenges in quantum communication is to develop techniques that can effectively preserve the unique properties of quantum mechanics during quantum information-processing tasks. Quantum Decoherence plays a significant role in this context and refers to the degradation of qubit properties within a quantum state due to unintended interactions with the environment. Quantum information processing heavily relies on maintaining coherent superposition states, which are particularly vulnerable to decoherence effects. These superposition states are essential for performing quantum computations and other quantum communication tasks. Therefore, when designing qubit systems, it is crucial to carefully consider and minimize the interactions with the environment to ensure the successful preparation and persistence of the desired superposition states.

Quantum decoherence is a fundamental phenomenon in quantum mechanics that occurs when a quantum system interacts with its environment, causing the loss of coherence. This occurs when the quantum phases and amplitudes of the system's wavefunction become entangled with the environment's degrees of freedom, effectively removing out quantum interference effects. This results in a mixed state, losing its unique quantum properties, and is a central challenge in quantum technologies, as the loss of coherence can degrade the performance of quantum computers, communication systems, and other devices. Factors contributing to quantum decoherence include interactions with other particles, temperature, and electromagnetic fields.

**Figure 10:**
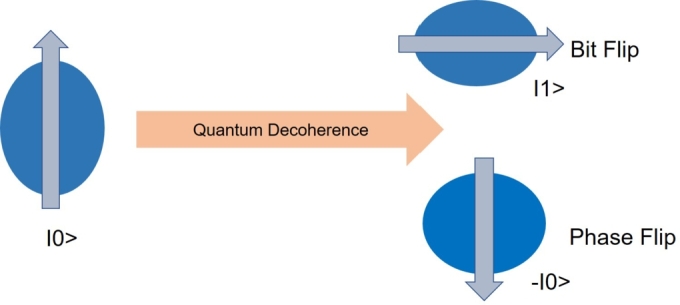
Effect of Quantum Decoherence on a qubit.

Quantum computer networks are inherently more complex than those needed for quantum key distribution and are susceptible to rapid decoherence [35], [36]. For quantum information processing systems, it is essential to build and encrypt the states properly. To perform all the necessary quantum logic operations, it is vital to slow down the rate of decoherence and minimize undesirable interactions with the environment [37]. Similar to classical binary symmetric channels, which produce a specific number of classical bit-flip errors within a given maximum Hamming weight, quantum Pauli channels generate errors from single-qubit errors and their combinations. As shown in Fig. 10, the two most common types of qubit errors are bit-flip errors and sign-flip errors. A bit-flip error for a single qubit is defined by the Pauli operator.

**Figure 11:**
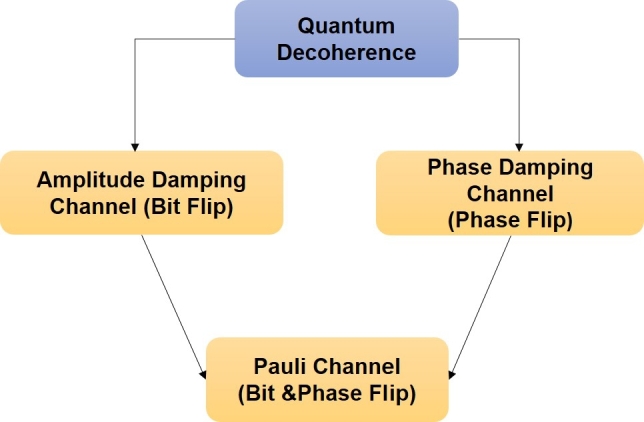
Quantum Decoherence Channels.

Qubits are said to have at least three different types of channel errors as depicted in Fig. 11, including Pauli-X bit-flip errors, Pauli-Z phase-flip errors, and Pauli-Y bit-flip and phase-flip errors [38]. In summary, interaction with the environment leads to the loss of entanglement, known as environmental decoherence. One instance of this is when the quantum system, such as a qubit, interacts with the outside world and loses energy. For example, a photon may emit spontaneously or get lost or absorbed as it propagates through optical fibers, causing the qubit's excited state to decay. This decoherence process can be effectively modeled using an amplitude-damping channel. Another type of environmental decoherence is dephasing or phase damping, where the quantum information is lost without any corresponding loss of energy. This can be caused by factors like photon scattering or disruptions in electronic states due to unwanted electrical charges. The Pauli channel combines the effects of both amplitude-damping and dephasing channels. It represents a more general type of decoherence, encompassing both energy loss and loss of quantum information without energy loss. Understanding and mitigating these types of decoherence are crucial in quantum communication and quantum computing applications to ensure the accuracy and reliability of quantum information processing. Efforts are being made to develop quantum error correction techniques and robust quantum systems to combat these decoherence effects and enable practical and efficient quantum technologies.

Multiple experiments have been conducted to demonstrate entanglement swapping from matter-light to matter-matter [39], [40]. In these trials, the initial step involves creating entanglement between photons and matter. Subsequently, the detection of one or two photons serves as an indication of entanglement between distant matters. An ongoing objective is to notably extend the distance across which quantum matter can be entangled to reach distances of 100 km or beyond. Such distances are crucial for establishing practical internode spacings, facilitating the development of expansive quantum networks. Optical fibers serve as a critical medium for achieving matter entanglement across extensive distances. Nevertheless, several challenges must be addressed before their widespread adoption. Firstly, matter systems emit photons at wavelengths that are highly absorbed by optical fibers, thus restricting the internode distance to only a few kilometers. A potential solution lies in single-photon quantum frequency conversion to the 1550 nm telecom C-band, which experiences minimal fiber transmission losses and is, therefore, an optimal choice for standard interfacing in quantum networking [41]. Secondly, preserving entanglement over long photonic channels poses another challenge. Decoherence processes, both uncontrolled ones acting on the photon during its travel and those affecting the quantum matter, can easily disrupt entanglement. For instance, attenuation of the entanglement-carrying photon signal, compounded by added photon noise from frequency conversion or dark counts of photon detectors, can overwhelm the signal over lossy waveguides. Additionally, the internode photon travel time sets a minimum coherence time for matter, which becomes significant over distances such as 50 km of optical fiber, at approximately 250 μs [41]. Thirdly, photon travel time introduces further challenges. The minimum time required to entangle remote matter in two locations equals the light travel time between them. With a 500 μs wait time over 50 km of optical fiber, the maximum attempt rate reaches only 2 kHz, necessitating a 500 μs wait to determine if an individual attempt at distributing remote entanglement succeeds. To achieve practical entanglement distribution rates, efforts can focus on increasing the probability of individual attempts succeeding or conducting multiple attempts in parallel. Based on the discussion above, Table 2 summarizes various quantities related to the decoherence channel.

**Table 2:** Review of Quantum Decoherence Channels.

Decoherence Channel	Description
Amplitude Damping	Describes the loss of coherence due to the system's interaction with its environment, leading to the decay of the quantum state's amplitudes.
Phase Damping	Represents the loss of information about the relative phase between quantum states, resulting from the system's coupling to a noisy environment.
Depolarizing	Describes a process where the quantum state undergoes random unitary transformations with certain probabilities, leading to the loss of coherence.
Phase Flip	Reflects the loss of coherence caused by random phase flips in the quantum state due to interactions with the environment.
Bit Flip	Represents the loss of coherence resulting from random bit flips (changes in the state's computational basis) due to environmental noise.
Generalized Amplitude Damping	Extends the amplitude damping model to account for additional effects such as dephasing and relaxation, providing a more comprehensive description of decoherence processes.

### 4.1 Decoherence model

Several important models that capture the dynamics of quantum decoherence are introduced in this subsection:

#### 4.1.1 Quantum master equation

The Quantum Master Equation, also known as the Lindblad equation, is a fundamental mathematical framework used to describe the dynamics of open quantum systems. These systems interact with their environment, leading to decoherence and the loss of quantum coherence. The equation provides a way to model the evolution of the density matrix, which characterizes the state of a quantum system. The general form of the Quantum Master Equation is:

(2) $$\frac{dt}{d\rho }=\frac{-i}{h}[H,\rho ]+\sum_{k}(L_{k}\rho L_{kt}-\frac{1}{2}(L_{kt}L_{k},\rho ))$$

Where:

• *p* is the density matrix representing the quantum state of the system.

• *H* is the system's Hamiltonian operator.

• $L_{k}$ are the Lindblad operators, describing the interaction of the system with its environment.

• *h* is the reduced Planck constant.

The first term in the equation represents the coherent evolution of the system governed by the Hamiltonian, while the second term accounts for the incoherent processes due to the interaction with the environment. The Lindblad operators model the effects of the environment, inducing both relaxation and dephasing of the quantum state.

The Quantum Master Equation plays a crucial role in understanding how open quantum systems evolve over time. It helps predict the behavior of quantum systems subject to decohering interactions, and it enables the study of various phenomena such as relaxation, decoherence, and quantum jumps. This equation is particularly relevant in fields like quantum optics, quantum information theory, and quantum thermodynamics, where the interaction with the environment cannot be ignored and where preserving quantum coherence is of paramount importance.

#### 4.1.2 Kraus operator

Kraus operators offer a mathematical foundation for characterizing quantum decoherence processes. In the context of quantum decoherence, Kraus operators reflect the probable consequences of a quantum system's interaction with its environment, which results in the loss of coherence and information. Mathematically, the evolution of the quantum state under decoherence can be described by a set of Kraus operators $E_{i}$, where each operator corresponds to a specific quantum operation that may occur due to the interaction with the environment. The evolution of the quantum state under decoherence can be expressed using the Kraus representation as [42]:

(3) $$\rho^{\prime }=\sum_{i}E_{i}\rho E_{i}^{†}$$

Where:

- *ρ* = the density matrix representing the initial state of the quantum system.

- $\rho^{\prime }$ = the density matrix representing the state after the decoherence process.

- $E_{i}$ = the Kraus operators, which form a complete set satisfying the completeness relation $\sum_{i}E_{i}^{†}E_{i}=I$, where *I* is the identity operator.

- $E_{i}^{†}$ = denotes the Hermitian conjugate of $E_{i}$.

Each Kraus operator $E_{i}$ describes a possible outcome of the decoherence process. These operators capture the effects of noise, errors, and interactions with the environment on the quantum state. The probabilities of different outcomes are determined by the specific characteristics of the decoherence process. For example, in the case of a depolarizing channel, which represents a common type of noise channel in quantum systems, the Kraus operators can be written as:

$$E_{1}=\sqrt{1-p}\;I,E_{2}=\sqrt{\frac{p}{3}}\;\sigma_{x},E_{3}=\sqrt{\frac{p}{3}}\;\sigma_{y},E_{4}=\sqrt{\frac{p}{3}}\;\sigma_{z}$$

Where:

*p* = the probability of an error occurring.

*I* = the identity operator.

$\sigma_{x}$, $\sigma_{y}$, and $\sigma_{z}$ are the Pauli matrices.

These Kraus operators describe the possible outcomes of the depolarizing channel, where the quantum state may remain unchanged (with probability 1−p) or undergo a Pauli-X, Pauli-Y, or Pauli-Z error (each with probability p/3).

In Markovian and non-Markovian research, Kraus operators are critical in distinguishing between memoryless and memory-retentive quantum dynamics. Markovian decoherence processes imply that the environment's influence on the quantum system depends purely on its current state, but non-Markovian processes display memory effects, where the system's previous history influences its current evolution. Kraus operators have been used by scientists to investigate Markovian and non-Markovian decoherence processes in a variety of quantum systems, such as trapped ions, quantum dots, and superconducting qubits. Within the field of quantum dots, scientists concentrate on developing novel materials to reduce interactions with the environment, improving device designs for better control, and integrating quantum dots with other quantum systems. Similar to this, improvements in hybrid system integration, control strategies, and trap design are critical in the field of ion traps. To research hybrid quantum dynamics, scientists construct traps with less interference from the environment, create complex control schemes to prevent decoherence, and investigate integration with other quantum platforms. To reduce coherence loss in the field of superconducting qubits, efforts are focused on improving qubit design, materials engineering, and noise suppression methods.

#### 4.1.3 Dephasing model

The dephasing model is a fundamental representation of a decoherence process that primarily affects the phase information of a quantum system. It describes the loss of coherence between different components of a superposition due to interactions with the environment, without altering the energy levels of the system. In the context of the density matrix formalism, the dephasing process is typically represented by Lindblad operators Lk that take the form of diagonal operators on a chosen basis. For a two-level quantum system (qubit), the dephasing Lindblad operators are:

(4) $$L_{1}=\sqrt{\gamma }\alpha_{z}$$

where *a*z is the Pauli-Z matrix representing the computational basis states. The parameter *γ* determines the rate at which the phase coherence is lost.

#### 4.1.4 Amplitude damping model

The amplitude-damping model captures a type of decoherence that involves the relaxation or decay of quantum amplitudes. It is particularly relevant in systems where quantum states can decay from an excited state to a ground state due to the interaction with the environment. In the density matrix formalism, the amplitude damping process is described using Lindblad operators Lk which are typically off-diagonal operators in a chosen basis. For a two-level quantum system, the amplitude-damping Lindblad operators are:

(5) $$L_{1}=\sqrt{\gamma }\alpha_{-}$$

(6) $$L_{2}=\sqrt{\gamma }\alpha_{+}$$

Here, $\alpha_{-}$ and $\alpha_{+}$ are the lowering and raising operators, respectively, which induce transitions between the excited and ground states. The parameter y determines the rate of the amplitude damping process.

#### 4.1.5 Caldeira-Leggett model

The Caldeira-Leggett model considers a quantum system of interest (often a two-level system or qubit) coupled to a bath of harmonic oscillators representing the environment. The interaction between the system and the bath leads to the transfer of energy and information between them. The model is described by a Hamiltonian that includes terms representing the system, the bath, and their coupling. The Caldeira-Leggett Hamiltonian takes the form:

(7) $$H=H_{s}+H_{b}+H_{sb}$$

where:

• $H_{s}$ = is the Hamiltonian of the system.

• $H_{b}$ = is the Hamiltonian of the bath.

• $H_{sb}$ = represents the interaction between the system and the bath.

The Caldeira-Leggett model demonstrates how the coupling between a quantum system and its environment leads to the suppression of quantum interference and the emergence of classical-like behavior. As the system interacts with the bath, its coherent superposition states gradually lose coherence due to the continuous exchange of energy and information with the environment. This process ultimately results in the selection of a classical-like state, commonly referred to as “pointer states,” which are more robust against the environment.

In a nutshell, interaction with the environment results in loss of entanglement, or environmental decoherence. In one such instance, interactions with the outside world cause the qubit (or quantum system) to expend energy. For instance, a photon may spontaneously emit or may be lost (or absorbed) as it travels through optical fibers, causing the excited state of the qubit to decay.

#### 4.1.6 Spin-boson model

The spin-boson model is a theoretical framework used to describe the interaction between a two-level quantum system (e.g., a spin-1/2 particle or a qubit) and a bath of harmonic oscillators (bosons), representing an environment. This model is essential for understanding phenomena such as decoherence, dissipation, and quantum phase transitions in open quantum systems. The Hamiltonian for the spin-boson model contains terms indicating the energy of the isolated quantum system, the energy of the bosonic modes in the environment, and the interaction between the system and the environment. The Hamiltonian for the spin-boson model can be written as [43]:

(8) $$\overset{ˆ}{H}=\frac{\omega_{0}}{2}{\overset{ˆ}{\sigma }}_{z}+\sum_{k}\omega_{k}{\overset{ˆ}{a}}_{k}^{†}{\overset{ˆ}{a}}_{k}+\frac{\sigma_{x}}{2}\sum_{k}(g_{k}{\overset{ˆ}{a}}_{k}^{†}+g_{k}^{⁎}{\overset{ˆ}{a}}_{k})$$

where:

• $\omega_{0}$ = is the energy splitting of the two-level system.

• ${\overset{ˆ}{\sigma }}_{z}$ = is the Pauli-Z matrix representing the two-level system.

• $\omega_{k}$ = are the frequencies of the bosonic modes in the environment.

• ${\overset{ˆ}{a}}_{k}^{†}{\overset{ˆ}{a}}_{k}$ = represents the coupling strength between the two-level system and the Bosonic modes.

The first term in the Hamiltonian describes the energy of the isolated two-level system, while the second term represents the energy of the bosonic modes in the environment. The third term describes the interaction between the two-level system and the environment, where $\sigma_{x}$ is the Pauli-X matrix representing the flip operator for the two-level system. The dynamics of the spin-boson model are governed by the Schrödinger equation, which describes how the state of the combined system (the two-level system plus the environment) evolves over time. The interaction between the two-level system and the environment leads to phenomena such as energy exchange, decoherence (loss of quantum coherence), and dissipation (energy loss).

Many experimental attempts have been made to understand the dynamics of the spin-boson model, which has provided essential insights into the behavior of open quantum systems. These studies use a variety of platforms, including superconducting qubits, trapped ions, and quantum dots, to investigate the interaction of a two-level quantum system with its bosonic mode environment. In a nutshell, interaction with the environment causes loss of entanglement, also known as environmental decoherence. In one such case, interactions with the outside environment lead the qubit (or quantum system) to lose energy. For example, a photon might spontaneously emit or be lost (or absorbed) as it passes via optical fibers, causing the excited state of the qubit to decay.

### 4.2 Mathematical description

Let us consider a qubit having two orthogonal basis states:

(9) |ψ〉=α|0〉+β|1〉

In this state, a qubit is in pure form which can be completely defined by its magnitude. Once the wave-function is expanded in the quantum basis relative to measurement performance, then the likelihood of obtaining measurement results is given by the square of the basis vector's coefficient. This is called Born's rule.

$$\text{If}\;\;\;\alpha =\frac{1}{\sqrt{2}}\;\text{Then}\;\;\;\alpha .\alpha^{⁎}=\frac{1}{2}=50\text{\%}\text{ probability}\;\alpha .\alpha^{⁎}+\beta .\beta^{⁎}=1$$

A qubit can be defined as:

$$|\psi \rangle =(1\sqrt{2})|0\rangle +(1\sqrt{2})|1\rangle$$

Adding a complex number $e^{i(\varphi )}|$ to state |1〉. Such a complex number doesn't change the probability of getting |0〉 and |1〉. Let us consider now the interaction of pure state qubit with the environment. The random phase will be added to each orthogonal state once interacted with environment:

$$|0\rangle =e^{i(\varphi_{0})}|0\rangle |1\rangle =e^{i(\varphi_{1})}|1\rangle$$

A qubit state becomes:

$$|\psi \rangle =(1\sqrt{2})|0\rangle +(1\sqrt{2})e^{i(\varphi )}|1\rangle$$

It can also be written as:

$$|\psi \rangle =(1\sqrt{2})e^{i(\varphi )}|0\rangle +(1\sqrt{2})|1\rangle$$

The *ϕ* is called as phase of the wave-function as it can decompose a complex number into sine and cosine:

$$e^{i(\varphi )}=\cos(\varphi )+j\cdot \sin(\varphi )$$

To measure the effect of quantum decoherence, calculating the density matrix is most important part. Density matrix denoted by P is given as:

$$P=|\psi \times \psi |=\left(\begin{array}{cc} \alpha \alpha^{⁎} & \alpha \beta^{⁎}\\ \beta \alpha^{⁎} & \beta \beta^{⁎} \end{array}\right)$$

For our case, the density matrix becomes:

$$P=\left(\begin{array}{cc} \frac{1}{2} & \frac{1}{2}e^{-i(\varphi )}\\ \frac{1}{2}e^{i(\varphi )} & \frac{1}{2} \end{array}\right)$$

According to the theory of quantum decoherence, a particle's phase actually changes every time it collides with another particle and measurements are the average of their random phases. For the complex number, every number with a maximum value of ‘1’ lies on the circle by varying the value of *ϕ* from 0 to 2*π*. The decoherence phenomenon lies not on the unit circle rather when averaged, it will converge to zero. After being pure initially, the states are changed to mixed states, where the off-diagonal terms have simply decayed away. More precisely, a pure qubit state may occur in the quantum channel as:

$$|\psi \rangle =\left(\begin{array}{cc} \left|a_{0}\right| & a_{0}a_{1}e^{i(\varphi_{0})}\\ a_{0}⁎a_{1}e^{i(\varphi_{1})} & \left|a_{1}\right| \end{array}\right)$$

After decoherence, the density matrix becomes:

$$P=\left(\begin{array}{cc} \frac{1}{2} & 0\\ 0 & \frac{1}{2} \end{array}\right)$$

**Figure 12:**
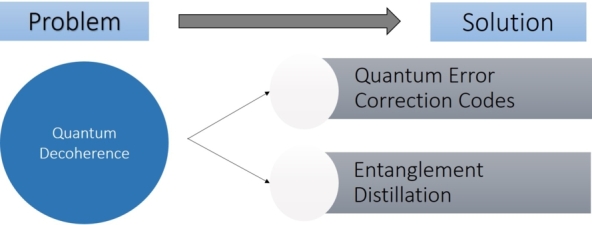
Quantum decoherence and its mitigation techniques.

Decoherence is the term used to describe a situation in which random changes completely disrupt the phase of the state. Coherent states have clearly defined phases and have the ability to interfere with one another. After decoherence, the matrix we obtained is no longer defined by any wave function. Due to this reason, quantum decoherence is called the collapse of the wave function. Primary methods for mitigation of quantum decoherence are Quantum Error Correction Codes (QECC) and Entanglement distillation, as shown in Fig. 12 [33].

## 5 Quantum error correction codes

To send quantum data from one quantum end node to another, quantum teleportation is the defining protocol of quantum communication. For the global quantum network to be established, quantum decoherence is the major impediment that needs to be addressed. This section will elaborate on the various techniques used for mitigation of quantum decoherence, as described in Fig. 12. Similar to classical communication, noise is also a major concern of the quantum channel. A quantum channel degraded by noise causes the transmitted qubit to flip its value depending upon certain probability, similar to the classical symmetric channel. A quantum majority vote can be counted to detect the one-bit flip error in the quantum process. For the three-qubit states, encoding can be done by:

$$|v\rangle \;\overset{\;}{\to }\;|vvv\rangle$$

**Figure 13:**
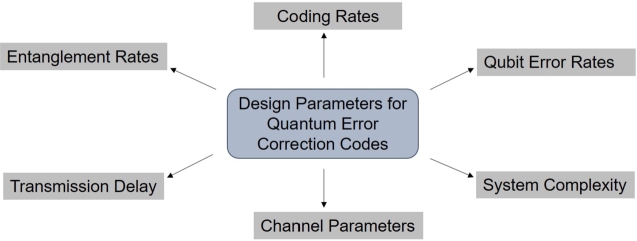
System parameters affecting the design of quantum codes [21].

To generate these code states or implement the encoding mechanism, it is adequate to set up a basic quantum circuit featuring a C-NOT gate controlling each of the initial binary ancillary components |0〉. Although the encoding of quantum information is a crucial part of any quantum error-correction scheme, quantum states are frequently vulnerable to a broader series of errors that may make superpositions of states encoded in this straightforward manner unrecoverable. Fig. 13 shows design parameters that need to be considered for designing the QECC.

**Figure 14:**
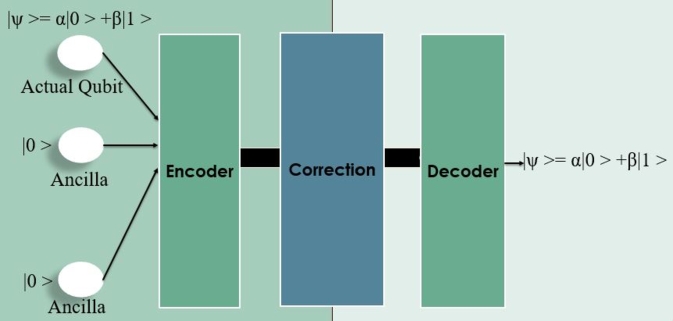
Three qubit error correction model.

A family of quantum error correction codes called three-qubit error correction codes is intended to shield quantum information from errors that might arise from decoherence or other forms of noise. These codes are under the larger category of quantum error correction, which tries to protect the integrity of quantum states in the face of outside disruptions. The bit-flip code, which can correct errors brought on by single-qubit bit-flip errors, is one well-known illustration of a three-qubit error correction code. The fundamentals of quantum error correction theory are illustrated through three-qubit error correction codes, which show how to encode and recover quantum information to mitigate the impact of errors. Despite their drawbacks, they act as the building blocks for more complicated and reliable quantum codes intended to defend quantum states as shown in Fig. 14.

### 5.1 Mathematical description of three qubit error code

As discussed, one of the most well-known three-qubit codes is the bit-flip code, which protects against single-bit flips. Here's a mathematical description of the bit-flip code:

#### 5.1.1 Encoding

The encoding process maps a single logical qubit |ψ〉=α|0〉+β|1〉 onto a three-qubit code space:

(10) $$|\psi \rangle \to |\psi_{0}\rangle =\alpha |000\rangle +\beta |111\rangle$$

The logical qubit can be in one of four states: |000〉, |100〉, |010〉, or |001〉, where each state corresponds to a different logical qubit value: $|0_{L}\rangle$, $|1_{L}\rangle$, $|2_{L}\rangle$, or $|3_{L}\rangle$, respectively.

#### 5.1.2 Error detection

The bit-flip code employs two parity check operators:

$$X_{1}X_{2}\;\text{and}\;X_{2}X_{3}$$

where $X_{i}$ denotes the Pauli *X* operator acting on the *i*-th qubit. These operators detect errors by measuring the parity of adjacent qubits.

- $X_{1}X_{2}$: This operator checks the parity of the first and second qubits. If both qubits are in the same state (both are either |0〉 or |1〉), the product $X_{1}X_{2}$ yields +1 (even parity). If the states are different (one is |0〉 and the other is |1〉), the product yields -1 (odd parity).

- $X_{2}X_{3}$: This operator checks the parity of the second and third qubits. Similar to $X_{1}X_{2}$, it yields +1 for even parity and −1 for odd parity based on the states of the second and third qubits.

By measuring the eigenvalues of these two parity check operators, we can obtain syndromes that indicate whether errors have occurred and, if so, which qubits are affected. If both parity check operators yield +1 (even parity), then no error has occurred. However, if one or both yield −1 (odd parity), it indicates that errors have occurred on the corresponding qubit(s). Measuring the eigenvalues of the parity check operators yields the syndromes. If both syndromes are +1, no error has occurred. If one or both syndromes are −1, an error has occurred on the corresponding qubit(s).

#### 5.1.3 Decoding

Based on the syndromes obtained, corrective operations are applied to the qubits to restore the original state. For example, if the first syndrome is −1, a *X* gate is applied to the first qubit to correct the error. To correct this error using an *X* gate, we apply the *X* gate to the first qubit.

If the initial state of the first qubit is |ψ〉, then the effect of applying the *X* gate can be represented mathematically as:

$$X|\psi \rangle =\left(\begin{array}{cc} 0 & 1\\ 1 & 0 \end{array}\right)\left(\begin{array}{c} a\\ b \end{array}\right)=\left(\begin{array}{c} b\\ a \end{array}\right)$$

where *a* and *b* are the probability amplitudes of the |0〉 and |1〉 states, respectively.

So, applying the *X* gate to the first qubit effectively flips the probability amplitudes of the |0〉 and |1〉 states. This operation corrects the error that occurred on the first qubit, restoring the qubit to its original state.

Effective quantum parallelism relies on high-visibility multi-qubit interference, which necessitates maintaining coherence, i.e., the period during which a qubit retains its state, throughout the computation. Quantum Error Correction Codes (QECC) address this by utilizing partial state measurements and entangled states to extract only error-related information while preserving the required state coherence, thereby mitigating issues arising from decoherence [37]. Information is stored in the quantum interaction between various modules of the complex system that implements the QECC. Even when a system's code state is affected by its environment, as long as it remains well correlated with other system components, the encoded data persists in this interaction and can still be recovered using error recovery techniques. Table 3 presents some examples of QECC developed between 1990 and 2010.

**Table 3:** Major achievements in quantum codes paradigm.

Year	Achievement
1990	Shor codes [44] CSS Codes [45] Quantum Stabilizer codes [46] Quantum BCH (QBCH) Codes [47] Quantum Reed Mullers Codes [48] Quantum Reed Solomon Codes [49]

2000	Quantum LPD Codes (QLPDC) [50] Quantum Convolutional Codes (QCC) [51] Quantum Turbo Codes (QTC) [52]

2010	Entanglement Assisted QCC [53] Entanglement Assisted Quantum Turbo Codes [54] Entanglement Assisted Polar Codes [55] Quantum IRCC [56]

The recovery of quantum states from decoherence was first proposed by Shor in 1995 [44], hence named Shor codes. It encodes a single qubit into nine qubits and can recover the quantum state correctly only if one qubit becomes decoherent. They proposed a method to encode quantum information into a subspace of the Hilbert space that is less susceptible to decoherence. By utilizing this encoding scheme, the authors demonstrated theoretically that the quantum information can be effectively shielded from decoherence, thereby enhancing the stability and reliability of quantum memory systems. Two qubit CNOT gate operation was carried out for the correction by Calderbank-Shor-Steane (CSS) code, which works until the errors induced are not significant [45]. This method uses the technique from the classical domain in the quantum domain. Quantum stabilizer codes create methods for examining quantum error-correcting codes and practice them to create an endless class of codes that saturate the quantum Hamming bound in 1996 [46]. These codes encode k = n-j-2 qubits in n = 2j qubits and correct t = 1 error, where t is the total length, k is the real qubits and n is the encoded qubits. Quantum Bose–Chaudhuri–Hocquenghem (BCH) codes were introduced which can encode quantum information effectively. It showed that four qubits were mandatory to encode one qubit and use a quantum erasure channel for this purpose [47]. As many classical error correction codes were incorporated in the quantum domain, quantum reed muller codes were also derived from classical reed muller codes [48]. Another QECC in this context was the Quantum Reed Solomon Code, whose construction is based on self-dual binary codes [49]. Due to limitations in computational powers, Quantum LDPC was not investigated largely until 2000. Quantum LDPC is constructed with the help of CSS code and addresses both bit flip and phase flip errors [50].

Quantum Convolutional codes (QCC) drew significant inspiration from classical convolutional codes, which have been widely used in classical communication under similar circumstances. The stabilizer formalism was employed to devise an explicit encoding circuit in one particular instance [51]. With the emergence of QCC, a quantum turbo code was constructed using its principles [52]. The encoder and decoders were designed by concatenating QCC. Similarly, Entanglement assisted-QCC was developed by leveraging CSS codes and incorporating binary convolutional codes for this purpose [53]. This approach was equally applicable to both classical and quantum communication. Additionally, entanglement assisted-quantum turbo codes were proposed, which served as a prerequisite for quantum turbo codes [54]. Several quantum encoders were designed based on entanglement, effectively performing error correction. Entanglement-assisted polar codes were also implemented to view quantum channels and effectively reduce the error rate through quantum decoding [55]. To explore the quantum-classical similarities, quantum irregular convolutional codes (QIRCC) were proposed, constructing 10 subcodes and utilizing them as an outer layer for convolutional codes [56]. These mentioned Quantum Error Correction Codes (QECC) are some of the methods implemented to safeguard qubits from the fragile nature of the environment.

Shor codes, a well-known type of quantum error-correcting codes, have drawn a lot of interest for their extraordinary capacity to fix particular quantum flaws. Shor codes, which are normally non-degenerate quantum error-correcting codes, are being looked into as degenerate codes that can correct all single quantum mistakes, according to a recent study [57]. By accepting single-qubit faults, this novel method intends to enhance the error-correcting power of Shor codes beyond the scope of their original design. Modern devices that accurately and efficiently transmit and disseminate quantum states are known as all-photonic quantum repeaters. By combining error-correcting capabilities based on the generalized Shor algorithm, the study presents a novel method to improve the transmission of quantum information across vast distances [57]. Building on the foundation of fault-tolerant quantum computation, Theodore J. Yoder [45] demonstrates that Bacon-Shor codes can effectively correct errors and enable error-resilient operations, offering a pathway to reliable quantum computation. In order to enhance the efficiency of the error correction process by dynamically adjusting the measurements based on real-time information, adaptive syndrome measurements for Shor-style error correction are proposed [59]. Adaptive techniques improve error mitigation, fault tolerance, and quantum computation reliability. A study [60] demonstrates Shor encoding on a trapped-ion quantum computer, achieving accuracy and advancement in error correction and trapped-ion computing capabilities making a significant step towards practical quantum error correction codes. It is summarized in the Table 4.

**Table 4:** Shor Codes.

Paper	Year	Contribution
Ahadiansyah [38]	2022	When used as degenerate codes, Shor codes eliminate all single quantum errors. This study focuses on the ability of Shor codes to handle different single-error conditions, revealing their versatility and efficiency in accomplishing robust quantum error correction.
Zhang [57]	2022	This research examines how Shor codes might improve the efficiency of quantum repeaters by reducing losses, hence advancing long-distance quantum communication.
Yoder [58]	2017	The paper offers a workable method for achieving fault-tolerant quantum computation using Bacon-Shor codes, opening the door for more durable quantum information processing methods.
Tansuwannont [59]	2023	The paper investigates enhanced ways for improving error correction efficiency, expanding the dependability of quantum information processing utilizing Shor-style error correction by introducing adaptivity into error correction protocols.
Nguyen [60]	2021	This demonstration shows that complicated quantum error correction codes may be executed by trapped-ion devices in a practical manner, advancing fault-tolerant quantum computers using Shor code.

In the quest for reliable quantum information processing, Steane's Code, another significant quantum error-correcting code, serves as a cornerstone. Rosie Cane et al.'s [61] suggests using Steane's Code to mitigate quantum-bit and quantum-gate defects brought on by decoherence. The study uses error-correcting methods to counteract the negative consequences of decoherence by fault-tolerantly encoding logical qubits. In order to enhance the fault tolerance in quantum computation, a study [62] explores Gate-Error-Resilient Quantum Steane codes by encoding qubits resilient to gate errors using concatenated Steane codes. This approach improves stability and reliability, highlighting its potential for practical quantum information processing. Entanglement-assisted quantum error correction codes are also deployed for the error correction. Research investigation intends to improve the performance of quantum error correcting codes supported by entanglement by utilizing the Steane encoding method proposed in [63]. An innovative way to expand the code space is introduced in this study, which could increase error-correction capabilities. Moreover, Error weight parities-based fault-tolerant quantum error correction enhances fault tolerance by adding parity checks based on error operators' weights, enabling effective error detection and correction, and proving its applicability in quantum systems [64]. For robust quantum error correction and more dependable quantum information processing, Steane codes present a possible route. A brief of it is summarized in Table 5.

**Table 5:** Steane Codes.

Paper	Year	Contribution
Cane, [61]	2020	This quantum error correction code is used in the study to examine efficient techniques for cutting mistakes in both quantum bits and quantum gates, improving the accuracy of quantum information processing with Steane code.
Cane, [62]	2020	The study explores the effectiveness of gate-error-resistant quantum Steane codes in reducing mistakes caused by defective gates, providing insights for developing more durable quantum error correction codes.
Galindo, [63]	2023	The paper explores the use of Steane code in expanding Entanglement-Assisted Quantum Error-Correcting Codes, aiming to improve efficiency and reliability in quantum error correction schemes.
Tansuwannont, [64]	2021	The paper investigates error weight parities-based fault-tolerant quantum error correction. Through the use of these parities, the research investigates methods for obtaining fault tolerance and boosting the dependability of quantum error correction procedures.

Quantum error-correcting codes (QECC) are a notion for direct quantum communication that entails encoding quantum information in a way that takes into account mistakes and noise during transmission. In this context, research study [65] direct quantum communications under realistic noisy entanglement. To simulate the impact of noise on quantum communication protocols, the technique models noise as depolarizing channels and highlights the significance of error correction approaches by showing how noise negatively impacts quantum information. Also, a paper introduces a one-step quantum secure direct communication protocol, utilizing entanglement and quantum gates for encoding and decoding messages [66]. This innovative approach eliminates the need for separate quantum channels and offers promising applications in cryptography and information transfer. Direct quantum communication can strive to become a reliable and safe way to communicate quantum information by minimizing the negative impacts of noise and decoherence, fostering improvements in secure communication and quantum networking paradigms as illustrated in Table 6.

**Table 6:** Direct Quantum Communication.

Paper	Year	Contribution
Chandra, [65]	2021	The investigation in this work centers on direct quantum communication with realistic noisy entanglement. This examines the difficulties of conveying quantum information across noisy channels and clarifies methods for achieving dependable direct quantum communication even when noise is present.
Sheng, [66]	2022	A one-step quantum secure direct communication mechanism is presented in the study. This presents a technique for securely and directly transmitting quantum information in a single step, aiding in the creation of safe and effective quantum communication protocols.

Another key component of quantum error correction, quantum stabilizer codes offer a strong framework for maintaining the integrity of quantum information. Recently, a paper [67] introduces a universal decoding strategy for Quantum Stabilizer Codes using classical guesswork. This approach bridges classical and quantum error correction techniques, expanding the toolbox for efficient and universal decoding in quantum information processing. Examination of fault-tolerant quantum gates with defects in topological stabilizer codes is also conducted [68]. It shows that topological stabilizer codes effectively mitigate defects, enabling fault-tolerant operations in imperfect physical implementations and contributing to advancing fault-tolerant quantum computation. New quantum stabilizer codes with enhanced parameters, inspired by RS and BCH codes are also proposed [69]. These codes improve performance and error correction by modifying the mathematical properties and structural features of classical error-correcting codes. Another study investigates coding non-binary Entanglement Unassisted and Assisted Stabilizer Codes, using encoding schemes based on stabilizer properties and non-binary entanglement [70]. The results demonstrate efficient procedures, expanding the toolkit for quantum error correction and entanglement-assisted communication protocols. The progress of quantum stabilizer codes will definitely be fueled by the synergy between theoretical advancements and real-world applications, opening the door for paradigm-shifting developments in the quantum field. Table 7 depicts the summarized version of these works.

**Table 7:** Quantum Stabilizer Codes.

Paper	Year	Contribution
Chandra, [67]	2023	It presents a universal decoding strategy for quantum stabilizer codes based on conventional guesses. This investigates techniques for effective decoding of quantum codes, enhancing error correction techniques, and enhancing the overall reliability of quantum information processing.
Webster, [68]	2020	This method explores fault-tolerant quantum gates with defects using topological stabilizer codes, aiming to develop robust gate operations while considering defects' impact, contributing to fault-tolerant quantum computation advancement.
Wang, [69]	2023	The paper presents new quantum stabilizer codes with improved parameters, enhancing performance through mathematical relationships between classical error-correcting codes and quantum stabilizer codes, potentially leading to more efficient and reliable quantum error correction strategies.
Nadkarni, [70]	2021	In this study, the encoding of stabilizer codes with and without nonbinary entanglement is examined. In order to construct effective and adaptable quantum error correcting codes that work on a variety of quantum platforms for encoding quantum stabilizer codes without the aid of entanglement.

Error correction codes have become the cornerstones of dependability in the complex world of quantum information, holding the key to releasing the full potential of quantum technology. These codes serve as the keystone connecting theoretical possibilities and actual implementations as we look towards the future. The persistent efforts to create, comprehend, and apply ever more complex error correction algorithms serve as the foundation for the pursuit of fault-tolerant quantum computers and secure quantum communication.

### 5.2 Entanglement distillation

The second method for protecting entangled pairs from the effects of decoherence is entanglement distillation, also known as Entanglement Purification. Entanglement distillation involves manipulating multiple copies of noisy, non-maximally entangled states to produce fewer copies with reduced noise. Entanglement distillation improves quantum decoherence by purifying and enhancing entangled states, using quantum error correction strategies to extract higher-quality entanglement. This interplay is crucial for maintaining reliability and fidelity in communication, computation, and information-processing tasks. This process finds applications in quantum teleportation and quantum computation [71] and was introduced in 1996 by Bennett et al. [72] to enhance communication over noisy quantum channels. Researchers such as Pan J-W et al. [73] and Reichle R et al. [74] demonstrated entanglement distillation for two different qubit sources: single photons and atoms, respectively.

**Figure 15:**
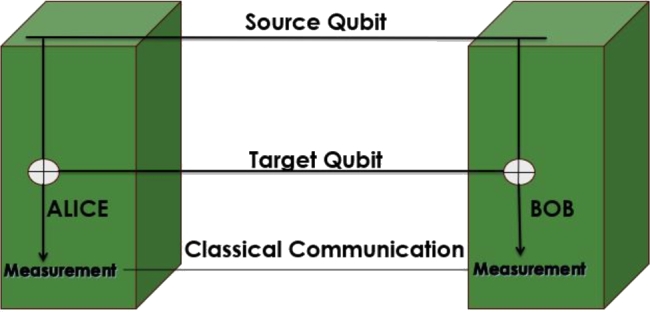
Entanglement distillation Model.

Quantum mechanics explains that qubits can exist in a superposition of states, combining their basis states. Entanglement between source and target qubits is generated by preparing a pair of Bell states, which are maximally entangled superpositions of two qubits. The source and target qubits interact in controlled ways, such as controlled gates, where the source qubit's state influences the target qubit's state. The combined state of the two qubits is then a Bell state or another entangled state. To confirm entanglement, both qubits are measured, with one qubit's state instantaneously determining the other's due to their entanglement. This process often involves quantum operations like controlled gates, CNOT gates, and complex circuits. The basis of entanglement generation using source and target qubits relies on creating specific quantum states where their properties are intertwined, affecting the state of the other as shown in Fig. 15.

#### 5.2.1 Mathematical description

Let's delve into the mathematical description of entanglement distillation:

The initial state of the entanglement distillation process is represented by an ensemble of mixed entangled states, denoted by *ρ*.

Mathematically, *ρ* is a density matrix that captures the statistical properties of the quantum system.

*Local Operation:*

In quantum mechanics, a density matrix *ρ* represents the state of a quantum system. It is a Hermitian, positive semidefinite matrix that captures all the statistical information about the system.

A local operation *E* acts independently on a subsystem of the quantum system. Mathematically, *E* is represented by a quantum channel, which is a completely positive and trace-preserving map. When *E* acts on the density matrix *ρ*, it transforms the state of the subsystem according to the rules of quantum mechanics. The action of *E* on *ρ* can be expressed as E(ρ), where *E* acts as a superoperator on the density matrix *ρ*. In terms of matrix multiplication, this operation can be represented as follows:

(11) $$E(\rho )=\sum_{k}E_{k}\rho E_{k}^{†}$$

Here, $E_{k}$ are the Kraus operators associated with the quantum channel *E*. These operators satisfy the completeness relation $\sum_{k}E_{k}^{†}E_{k}=I$, where *I* is the identity operator. The action of *E* on *ρ* results in a new density matrix E(ρ), which represents the state of the subsystem after the local operation *E* has been applied. This operation allows us to manipulate the state of individual subsystems of the quantum system, enabling various quantum information processing tasks such as error correction, state preparation, and measurement.

*Entanglement swapping:*

Entanglement swapping involves performing Bell measurements on pairs of qubits and applying conditional unitary operations. Mathematically, entanglement swapping can be expressed as:

$$\rho_{12}^{\prime }=B_{12}(\rho_{12}⊗\rho_{34})B_{12}^{†}\;\rho_{34}^{\prime }=U_{ab}(\rho_{12}⊗\rho_{34})U_{ab}^{†},$$

where $B_{12}$ represents a Bell measurement and $U_{ab}$ represents a conditional unitary operation.

*Error Correction:*

Error correction techniques involve applying corrective operations to mitigate noise and decoherence effects. Corrective operations can be represented by quantum gates or unitary matrices.

If *C* represents a corrective operation, the corrected state $\rho^{\prime }$ can be expressed as:

(12) $$\rho^{\prime }=C(\rho )$$

- Entanglement measures quantify the degree of entanglement present in the purified state. For example, if E(ρ) represents the entanglement measure of state *ρ*, it can be expressed as E(ρ).

Mathematical operations are utilized in each phase of the entanglement distillation process to modify the quantum state represented by the density matrix. We may improve entangled states and increase their degree of entanglement by using these techniques iteratively.

Entanglement distillation can be classified into two types: Probabilistic Entanglement Distillation and Deterministic Entanglement Distillation. Probabilistic protocols require two nonlocal parties and two similar pairs of noisy entangled states. To determine whether purification was successful, they measured the second copy after executing controlled-not (CNOT) operations or similar operations. If successful, a high-fidelity entangled pair is accepted; otherwise, both entangled state pairs are discarded. These protocols can eliminate bit-flip errors in a single round, and any remaining errors can be addressed in subsequent rounds using Hadamard operations. On the other hand, Deterministic Entanglement Distillation leverages Hyper-entanglement to deterministically purify fragile polarization entanglement in noisy environments [75]. Hyperentanglement involves the simultaneous entanglement of two or more degrees of freedom, such as position-momentum, and energy-time, in a multipartite quantum state [76].

The goal of entanglement distillation research is to create methods for improving and purifying entangled states that are impacted by noise, decoherence, and flaws. In research, machine learning approaches are used to optimize the distillation process while taking into account the limitations of noisy classical communication channels. The paper explores learning quantum entanglement distillation utilizing noisy classical communications [77]. It also shows how these techniques improve entanglement distillation's effectiveness, allowing for the production of highly effective entangled states. Using quantum memories optimistic entanglement purification focusing on protocols that improve entanglement despite scarcity is explored [78]. The study demonstrates the viability of this approach, offering insights into maximizing the benefits of limited memory resources for entanglement purification, impacting quantum communication and information processing. Investigation on experimental single-copy entanglement distillation, using entanglement swapping and post-selection techniques is performed [79]. The protocol extracts higher-fidelity entanglement from single-copy states, making it a significant advancement in quantum communication and practical for resource-limited scenarios. Another paper investigates long-distance entanglement purification strategies for quantum communication, focusing on protocols to purify entangled states across extended distances [80]. It uses innovative techniques and theoretical frameworks to mitigate noise and decoherence, preserving entanglement fidelity. Table 8 shows some entanglement distillation work discussed earlier.

**Table 8:** Study of Entanglement Distillation.

Paper	Year	Contribution
Chittoor, [77]	2023	In order to increase the efficiency and effectiveness of quantum error correction procedures in the face of classical communication noise, the study investigates ways to strengthen entanglement distillation processes by combining machine learning approaches.
Mobayenjarihani, [78]	2021	This study presents a method for improving the quality of entangled states with little memory overhead, which could eventually lead to more effective and useful entanglement purification methods for quantum computation and communication using small number of quantum memories.
Ecker, [79]	2021	The study demonstrates experimental single-copy entanglement distillation, enhancing entanglement quality in a single iteration, contributing to efficient quantum error correction strategies and practical implementation of entanglement-based quantum applications.
Hu, Xiao-Min, [80]	2021	The paper explores long-distance entanglement purification techniques for quantum communication, aiming to improve quality, address noise and decoherence issues, and develop reliable protocols.

Deterministic entanglement distillation enhances state quality with certainty, unlike probabilistic methods that may yield higher-quality states with probability. Protocols use deterministic operations like entanglement swapping and local operations without random outcomes. In this context, A methodology of work improves entangled polarization states' quality using deterministic operations, demonstrating successful purification through a single step, contributing to advancements in deterministic entanglement purification techniques [75]. For quantum networks, the study explores deterministic operations for controlled entanglement across network nodes using series-parallel architectures, demonstrating successful distribution and advancing quantum communication and networking feasibility [81]. A paper highlights recent advancements in the field of quantum entanglement purification [82]. Another way is Deterministic coherence distillation -a deterministic approach to enhance quantum coherence, focusing on designing protocols that amplify quantum states' coherence while minimizing probabilistic processes [83]. This approach contributes to the stability and reliability of quantum technologies, advancing information science and enabling robust applications in computation, communication, and sensing. A brief of it is shown in Table 9.

**Table 9:** Deterministic Entanglement Distillation.

Paper	Year	Contribution
Huang, [75]	2022	An experimental one-step deterministic polarization entanglement purification process is presented in the study. This work contributes to the development of practical and effective techniques for improving the quality of entangled states in quantum communication and information processing by demonstrating the viability of high-quality entanglement purification in a single step.
Meng, [81]	2023	The paper explores deterministic entanglement distribution on series-parallel quantum networks, aiming to improve the practical implementation of this distribution for quantum communication and networking.
Yan, [82]	2023	The paper discusses recent advancements in quantum entanglement purification techniques, highlighting their potential to improve the quality and fidelity of entangled states, thereby enhancing quantum communication and computation efficiency.
Liu, C. L, [83]	2019	Deterministic coherence distillation is a technique that systematically enhances quantum coherence through incremental improvements in quantum state coherence. This approach contributes to the advancement of quantum information science and technology by employing deterministic operations and refining coherence distillation processes.

Various techniques, including the use of photons, atomic ensembles, and hybrid approaches, which combine both techniques, are used for entanglement distillation. Recently, a paper presented a method for achieving entanglement distillation between atomic ensembles using high-fidelity spin operations [84]. The methodology improves entanglement content by utilizing precise spin manipulations by showing progress in successful distillation, highlighting the potential of accurate spin operations for enhancing entanglement-based quantum technologies' robustness and efficiency. Another technique is the photon, which is investigated by using polarization entanglement establishment in noisy channels, addressing challenges in maintaining quality and transmitting photons [85]. These are employed to mitigate noise effects and enhance entanglement fidelity, contributing to quantum communication advancements. Another method described in the research uses hybrid entanglement switching and variational distillation techniques to entangle remote microwave quantum computers. In order to improve the quality of shared quantum states, the study intends to establish entanglement between remote quantum processors in the microwave range. The outcomes indicate effective entanglement, illuminating a possible strategy for coupling quantum processors over considerable distances [86] as summarized in Table 10.

**Table 10:** Experimental realizations of entanglement distillation.

Paper	Year	Contribution
Liu, [84]	2022	By utilizing high-fidelity spin processes, the research focuses on entanglement distillation between atomic ensembles. In order to improve the quality of entanglement inside atomic systems and open the door to more dependable and effective quantum communication and computation technologies.
Ecker, [85]	2023	The study looks at how noisy polarization channels can create polarization entanglement between distant places. In order to enhance trustworthy quantum communication protocols, it examines obstacles to attaining reliable entanglement distribution under the effect of noise.
Zhang, [86]	2022	Entangling remote microwave quantum computers involves initializing qubits, establishing entanglement via entanglement swapping, and refining entanglement quality through variational distillation. This process aims to correlate quantum states across distant systems.

A vital link between the complex world of quantum phenomena and the practical requirements of quantum technology is provided by entanglement distillation. The impressive advancements in distillation techniques have highlighted the possibility of producing dependable entanglement in the presence of noise and flaws. In the future, exploiting the synergy between theoretical progress and experimental innovation will lead to improved distillation techniques. The voyage of entanglement distillation has continued to be both a monument to human creativity and a promising path for realizing the revolutionary promise of quantum technology as the field of quantum information science expands. Based on the discussed approaches, a comparative analysis will be conducted in the upcoming section.

## 6 Comparative analysis of mitigation techniques

**Figure 16:**
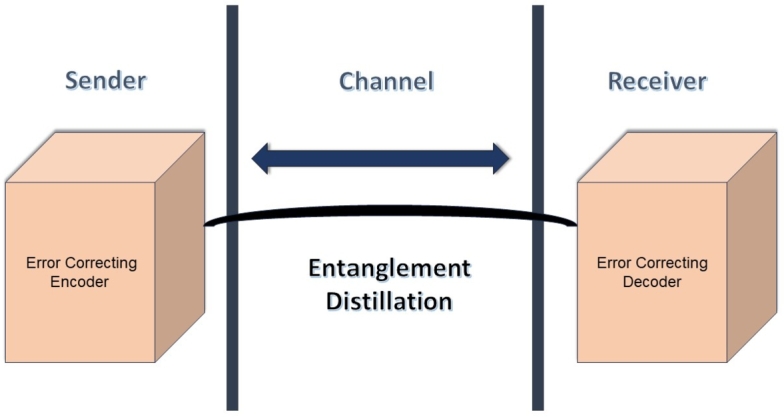
Quantum Decoherence Methods.

Quantum decoherence is a significant obstacle in quantum teleportation protocols, prompting extensive research in Quantum Error Correction Codes (QECCs) that draw from classical error correction codes to handle quantum errors. Unlike QECCs, entanglement distillation lacks classical counterparts due to the fundamental differences between quantum and classical mechanics. In quantum communication systems, QECCs typically serve as a pre-processing block to safeguard quantum information during transmission. On the other hand, entanglement distillation acts as an intermediary between the pre and post-processing stages, as shown in Fig. 16, aiming to improve the quality of entangled states. This process plays a crucial role in various quantum protocols, including quantum teleportation. Both QECCs and entanglement distillation are crucial components in quantum communication, addressing distinct challenges related to quantum errors and decoherence.

**Figure 17:**
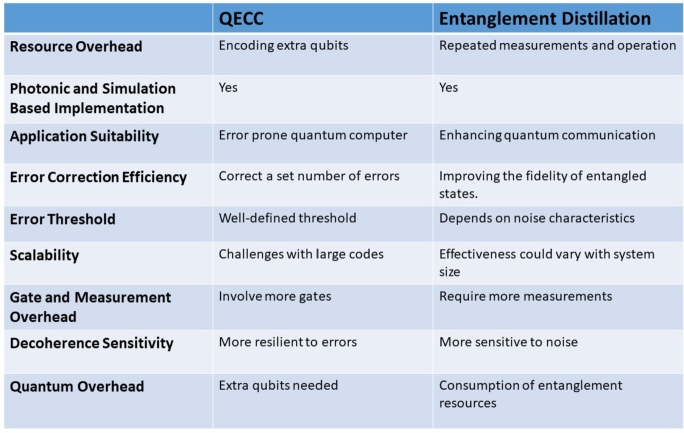
Comparative analysis of QECC and Entanglement Distillation.

Quantum Error Correction Codes (QECCs) add redundancy to actual quantum information or qubits, while entanglement distillation involves converting noisy entangled copies into a system with less noise. Fig. 17 compares the reviewed techniques. QECCs come with a cost in terms of the number of physical qubits needed to encode a logical qubit. This overhead is influenced by properties such as the number of encoded qubits, distance, and error tolerance. More extensive error correction generally requires more physical qubits, leading to increased hardware requirements. These codes also have an error threshold, representing the maximum error rate that allows reliable quantum computations. Codes with higher error thresholds can tolerate a larger error rate but typically necessitate more qubits and additional resources. Scalability is a critical concern for QECCs, as it becomes increasingly challenging to scale error correction codes as quantum systems grow in size. Researchers are actively working on designing codes that can efficiently correct errors on large-scale quantum architectures. Entanglement distillation also introduces overhead, as it requires multiple copies of entangled states to achieve the desired level of purity. The amount of overhead depends on the specific distillation protocol and the initial quality of the entangled states.

The main purpose of entanglement distillation is to increase the fidelity or purity of entangled states. Distillation protocols are designed to improve the quality of entanglement by removing noise and unwanted correlations. This results in high-fidelity entangled states, which are crucial for more robust and reliable quantum information processing. Quantum error correction codes, on the other hand, primarily focus on protecting quantum information from errors and decoherence. They employ encoding and decoding schemes to detect and correct errors, ensuring the reliability of quantum computations. While both error correction codes and entanglement distillation aim to improve quantum information processing, they have different roles. Error correction codes protect quantum information from errors, while entanglement distillation enhances the quality of entangled states. Both error correction codes and entanglement distillation come with overhead in terms of resources required. Error correction codes require additional physical qubits to encode logical qubits, and entanglement distillation requires multiple copies of entangled states. Another important difference is that quantum error correction codes have an associated error threshold, which determines the maximum error rate that can be tolerated while still enabling reliable quantum computations. Entanglement distillation aims to improve the fidelity of entangled states but does not have a strict error threshold.

Error correction codes and entanglement distillation are closely interconnected in many quantum computing systems. High-quality entangled states play a crucial role in effective error correction, as certain error correction codes rely on specific entangled states as resources. To develop error-resilient quantum algorithms that are less sensitive to decoherence, researchers are exploring strategies to tolerate and mitigate the effects of noise and errors. By designing algorithms that can withstand and minimize the impact of decoherence, the reliability of quantum computations can be improved. Advancements in quantum error correction codes and fault-tolerant techniques are essential in the fight against decoherence. Efforts are being made to develop more efficient and scalable codes, optimize error correction procedures, and explore novel strategies tailored to specific quantum hardware architectures. Improving quantum hardware technologies, such as qubit coherence times, gate fidelities, and noise reduction techniques, is crucial for mitigating decoherence. Researchers are working on developing new materials, fabrication methods, and control mechanisms to enhance the stability and coherence of quantum systems. Quantum error mitigation techniques aim to reduce the impact of errors and decoherence without necessarily requiring full error correction. These methods leverage classical post-processing and machine learning algorithms to estimate and correct errors, enabling more reliable quantum computations in the presence of noise. Advancements in quantum control techniques, such as optimal control theory and adaptive feedback control, can also contribute to mitigating decoherence. These techniques enable precise manipulation and protection of quantum states, leading to better coherence preservation and error suppression. Overall, research in these areas will play a vital role in advancing quantum computing and overcoming the challenges posed by decoherence.

**Figure 18:**
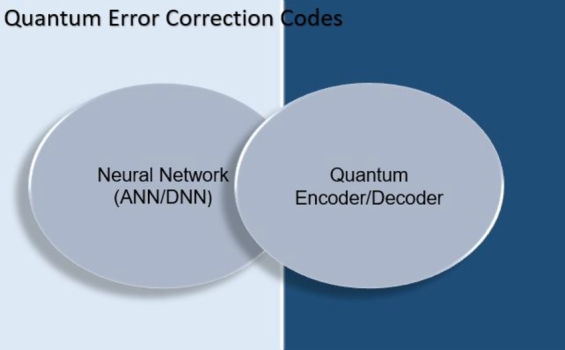
Futuristic quantum error correction methods.

Indeed, machine learning, particularly neural networks, has shown great promise in various domains, and it can also be employed to enhance quantum error correction codes (QECs) as shown in Fig. 18. Neural networks can be utilized for the encoding and decoding process in QECs, which involves identifying and correcting errors in received quantum states using the code's redundancy. By training neural networks, they can learn the decoding rules and patterns of errors, making them efficient in error correction tasks within noisy quantum systems. The neural network takes the noisy quantum state as input and generates the corrected state as output. Moreover, neural networks can serve as powerful tools for predicting and estimating errors in quantum systems. By analyzing the characteristics of a given quantum state and its evolution over time, neural networks can learn to predict the most likely types and locations of errors that might occur. This information can then be used to design effective error correction strategies, ultimately improving the overall performance of the QEC code. The integration of neural networks with quantum error correction holds great potential for advancing quantum computing technologies and making them more robust against decoherence and errors. As machine learning continues to advance, it is likely to play an increasingly significant role in various aspects of quantum information processing.

Neural networks have shown promising potential in optimizing and designing quantum error correction codes. By training neural networks on a dataset of input quantum states and their corresponding error patterns, they can learn to identify optimal or near-optimal code structures and parameters that maximize error correction capabilities. This approach can lead to the discovery of new and more efficient quantum error correction codes, as well as improvements to existing ones. However, it is important to acknowledge that the field of neural networks for quantum error correction is still in its early stages, and there are ongoing research efforts to explore the full range of applications and limitations of these techniques. The practical implementation of neural networks for quantum error correction would heavily depend on the availability of large-scale, fault-tolerant quantum computers, as these methods typically require significant computational resources. Despite the current challenges, the combination of neural networks and quantum error correction holds great promise for advancing the field of quantum computing. As quantum technologies continue to develop, neural networks could play a crucial role in enhancing the performance of quantum error correction codes and making quantum computation more robust and efficient. Further research and experimental validation are necessary to fully realize the potential of neural networks in quantum error correction and beyond.

### 6.1 Design challenges

**Figure 19:**
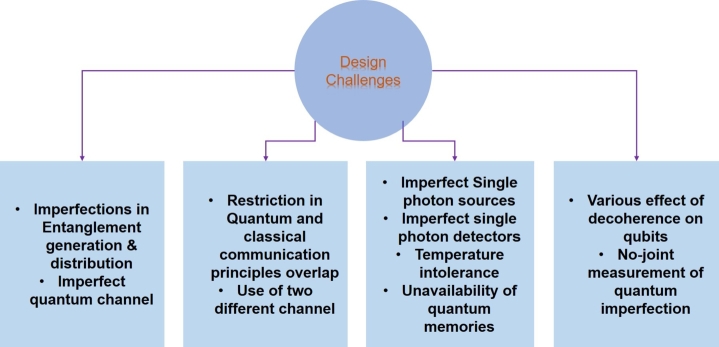
Imperfections in Quantum communication process.

In this section, we discuss the open-ended problems and difficulties encountered in realizing quantum internet. Some of the major issues in the quantum domain for full realization of full-scale quantum networks are discussed in the following text and summarized in Fig. 19.

#### 6.1.1 Imperfections in quantum teleportation

Just like classical communication, quantum communication is also affected by imperfections. Quantum decoherence, as discussed in Section 3, is one of the serious obstacles in quantum communication. Imperfections can occur in various aspects of quantum communication, such as in the pre/post-processing of quantum states, the quantum channel itself, and the loss of qubit states due to interactions with the environment. These imperfections pose significant obstacles to achieving perfect quantum teleportation. Another challenge lies in quantum gate operations, which process and transmit qubits. Limitations in photonic devices and other quantum components can introduce errors and imperfections in the quantum operations. Moreover, these quantum imperfections are multiplicative in nature, meaning that their impact can accumulate and become more significant over time, resembling the classical fading effect rather than the typical additional noise caused by the Brownian motion of electrons [87]. Despite these challenges, there is a lot of ongoing research aimed at advancing photon, atom, and solid-state experiments, as well as applications in quantum communication and computation [88]. Researchers are continuously exploring novel techniques to mitigate and correct quantum imperfections, such as quantum error correction codes and quantum fault-tolerant strategies. As our understanding of quantum systems and technologies improves, we can expect significant progress in overcoming these imperfections and achieving more reliable and efficient quantum communication and computation systems.

#### 6.1.2 Classical-quantum integration

A fully functional quantum network necessitates a seamless integration between quantum and classical channels. However, the non-cloning theorem, as explained in section 1, prohibits the direct measurement of qubits. As a result, assuming a straightforward implementation between classical communication encoders (decoders) and their quantum counterparts is not possible. To enable qubit teleportation and create desired nodes with maximum entanglement, it becomes essential for quantum and classical communication models to support each other. In recent times, researchers have proposed a quantum network structure centered around entanglement [89]. This model illustrates how the synergy between quantum and classical elements will lead to a comprehensive Quantum Information (QI) structure through the use of quantum repeaters.

#### 6.1.3 Hardware limitations

The physical interaction of qubits with the outside world is the main cause of quantum decoherence. However, the limitations of photonic hardware devices, such as single photon sources and detectors, also carry significant implications. Over the past decade, integrated quantum photonics has made significant strides, evolving into a multifaceted platform that promises to drive the progress of future quantum technologies and applications. By adopting wafer-scale fabrication processes, the deployment of integrated photonic platforms and systems is expected to continue expanding throughout the next decade, revolutionizing quantum communication and quantum information processing [90]. Nevertheless, several intriguing technological challenges need to be addressed before achieving complete integration. One such problem is the control of photons at the cryogenic temperatures required for detector operations. Solving these issues will play a pivotal role in unlocking the full potential of integrated quantum photonics.

#### 6.1.4 Joint modelling of imperfections

Even though the accurate simulation of quantum-domain issues is of utmost importance, there remains a challenge in achieving this goal and effectively capturing the consequences of the various imperfections in the quantum teleportation process. As these imperfections affect qubits differently, it becomes crucial to have a comprehensive model that can measure their combined effects. Different models are employed to illustrate various damping issues, such as phase, y-z, and combined y-z. To overcome these challenges, the use of a composite quantum imperfections model becomes essential. This model will facilitate the attainment of more precise results concerning quantum entanglement loss and quantum errors. By considering the diverse imperfections as a whole, researchers can enhance the understanding and analysis of quantum teleportation processes, leading to more robust and accurate quantum technologies.

## 7 Future quantum Internet technology

Table 11 summarizes the future applications of Quantum networks, which are further discussed in the following sections.

**Table 11:** Future Applications of Quantum Networks.

Application	Description
Quantum Cryptography	Utilizes the principles of quantum mechanics to enable secure communication, ensuring that any attempt to eavesdrop on the communication will be detected.
Quantum Internet	A global network infrastructure that enables quantum communication and quantum computing across vast distances, revolutionizing fields like finance, healthcare, and logistics.
Quantum Sensor Networks	Utilizes entangled quantum states to achieve unprecedented levels of sensitivity and precision in sensing and monitoring applications, such as early detection of diseases and environmental monitoring.
Quantum Cloud Computing	Leverages distributed quantum computing resources accessible via the internet, offering unparalleled computational power for complex simulations, cryptography, and optimization problems.
Quantum Machine Learning	Harnesses quantum algorithms to train and infer machine learning models, enabling breakthroughs in pattern recognition, data analysis, and optimization tasks.
Quantum Simulation	Allows the simulation of complex quantum systems, offering insights into material properties, chemical reactions, and fundamental physics phenomena that are difficult to study experimentally.
Quantum Enhanced Sensing	Enhances detection capabilities in various domains, such as medical imaging for early disease diagnosis, environmental monitoring for pollution detection, and defense applications for threat detection.
Quantum Communication Satellites	Enables secure global communication by deploying satellite-based quantum communication links, offering unbreakable encryption for government, military, and financial applications.

### 7.1 Quantum machine learning

Quantum Information (QI) has become a prominent research field, attracting considerable attention for its complete network topology. Beyond that, quantum communication holds enormous potential in various applications, including clock synchronization [91], blind computing (BC) [92], and quantum machine learning (QML) [93]. QML is a method that combines quantum mechanics principles with machine learning algorithms, implementing machine learning concepts in the quantum domain. Additionally, deep learning techniques can leverage specialized hardware like quantum annealers to enhance performance. Researchers have extensively explored the concept of a quantum artificial network [94], and recent experiments by Simões et al. [95] involved quantum support vector machines (QSVM) and quantum neural networks (QNN). It is predicted that there will be a substantial increase in the accessibility of quantum data in the near future, which logically follows from the use of available quantum computers. As more quantum issues are solved and quantum simulations are performed [96], an abundance of quantum data sets is expected to emerge.

### 7.2 Software defined networks

To enhance network functionalities and enable better control and management, programmable network configurations are employed, commonly referred to as Software-Defined Networks (SDNs). While SDNs are extensively used in classical communication, their utilization is steadily increasing in the quantum domain. A recent example is the Illinois Express Quantum Network (IEQNET) architecture, which utilizes SDN technology to facilitate conventional wavelength routing and assignment between Q-Nodes [97]. This illustrates how quantum networks are integrating classical network principles, showcasing the ongoing interdisciplinary efforts in this field. Another instance is the Madrid quantum network, where SDN has been successfully demonstrated, effectively integrating both quantum and classical channels [98]. This underlines the critical role SDN plays in upcoming quantum networks, paving the way for the future quantum internet.

### 7.3 Quantum enabled 6G communication

With the advent of 5G cellular networks, research on the development of beyond 5G (B5G) and 6G technologies is progressing rapidly. Similarly, in the quantum domain, Quantum Information Processing (QIP) is playing a crucial role in advancing future quantum 6G communication [99]. It is anticipated that QIP will contribute significantly to the architecture and capabilities of future 6G wireless networks.

The integration of quantum computing (QC), machine learning (ML), and quantum machine learning (QML) with communication terminologies is considered a key enabler for 6G technologies [100]. Recent advancements in quantum photonic computing have shown the feasibility of room-temperature-based quantum hardware. Moreover, the well-established infrastructure supporting fiber-optic-based communication equipment can be readily interfaced with photonic-based quantum hardware, offering a viable pathway toward the realization of 6G in the future.

## 8 Conclusions

Quantum Information (QI) has shown remarkable potential as a future quantum technology, offering ultra-secure communication and immense computational power. However, one of the major hindrances faced in this field is quantum decoherence, which degrades qubits' characteristics and pushes them into the classical domain. This poses a significant challenge for long-distance quantum communication, hindering the progress of quantum internet technologies. Despite this challenge, the captivating phenomenon of quantum entanglement has significantly advanced the domain of quantum computing. Leading companies and giants have shown a growing interest in this mysterious yet fascinating sector, recognizing the immense potential in various quantum domains, including quantum internet. To address the challenges posed by quantum decoherence, techniques like entanglement purification or distillation and quantum error correction codes are being extensively researched. These methods aim to mitigate the impacts of imperfections and enhance the reliability of quantum communication. Photonic innovation has emerged as a fundamental facilitator for the development of quantum internet on the hardware platform. However, realizing the full potential of quantum internet requires the collaboration and contribution of multiple disciplines, including network technologies, electronics and communication systems engineering, and quantum physics. This multidisciplinary endeavor will play a pivotal role in positioning imminent quantum networks for success and enabling the realization of a truly interconnected quantum internet.

In this paper, we have delved into the foundational concepts of quantum mechanics and the mathematical formulations that serve as the basis for quantum computing and the quantum network. Additionally, we provided an overview of quantum communication, focusing on the fundamental process of quantum teleportation. Throughout the discussion, we highlighted the key obstacle of quantum decoherence, which poses challenges for quantum information processing. To tackle the issue of quantum decoherence, we conducted a comparative analysis of various methods, primarily Quantum Error Correction Codes (QECC) and entanglement distillation, which aim to reduce its detrimental effects. Finally, we explored several technological challenges and potential research applications in the realm of the quantum network. In conclusion, it is evident that addressing the difficulties and obstacles related to the quantum network will require extensive frontier research. However, as the quantum internet paves the way for the future Internet, the excitement and allure of contributing to this burgeoning field of study are truly irresistible. Embracing and overcoming these challenges will undoubtedly lead to groundbreaking advancements in the field of quantum technologies, opening up new possibilities and transforming the landscape of communication and computation.

## CRediT authorship contribution statement

**Muhammad Annas Khan:** Writing – original draft. **Salman Ghafoor:** Writing – review & editing, Supervision. **Syed Mohammad Hassan Zaidi:** Conceptualization. **Haibat Khan:** Methodology. **Arsalan Ahmad:** Methodology.

## Declaration of Competing Interest

The authors declare that they have no known competing financial interests or personal relationships that could have appeared to influence the work reported in this paper.

## Data Availability

Not applicable.
